# Early detection of esophageal second primary tumors using Lugol chromoendoscopy in patients with head and neck cancer: A systematic review and meta‐analysis

**DOI:** 10.1002/hed.25548

**Published:** 2018-12-28

**Authors:** Oisín Bugter, Steffi E. M. van de Ven, Jose A. Hardillo, Marco J. Bruno, Arjun D. Koch, Robert J. Baatenburg de Jong

**Affiliations:** ^1^ Department of Otorhinolaryngology and Head and Neck Surgery Erasmus MC Cancer Institute Rotterdam The Netherlands; ^2^ Department of Gastroenterology and Hepatology Erasmus MC Cancer Institute Rotterdam The Netherlands

**Keywords:** early detection, esophageal cancer, head and neck cancer, Lugol chromoendoscopy, second primary tumor

## Abstract

**Background:**

Early detection of esophageal secondary primary tumors (SPTs) in head and neck squamous cell carcinoma (HNSCC) patients could increase patient survival. The purpose of this study was to determine the diagnostic yield of esophageal SPTs using Lugol chromoendoscopy.

**Methods:**

A systematic review of all available databases was performed to find all Lugol chromoendoscopy screening studies.

**Results:**

Fifteen studies with a total of 3386 patients were included. The average yield of esophageal‐SPTs in patients with HNSCC was 15%. The prevalence was the highest for patients with an index hypopharyngeal (28%) or oropharyngeal (14%) tumor. The esophageal‐SPTs were classified as high‐grade dysplasia in 49% of the cases and as invasive carcinoma's in 51%.

**Conclusion:**

Our results show that 15% of the patients with HNSCC that underwent Lugol chromoendoscopy were diagnosed with an esophageal‐SPT. Based on these results there is enough evidence to perform Lugol chromoendoscopy, especially in an Asian patient population.

## 1 INTRODUCTION

Part of the mortality of patients treated for head and neck squamous cell carcinoma (HNSCC) is caused by the occurrence of second primary tumors (SPTs).^1^ Risk factors for their development include alcohol and tobacco use, age, and the sub‐location of the index tumor (eg, hypopharynx).^2^ Most SPTs in patients with HNSCC occur in the head and neck region, esophagus, and lungs.^1^, ^3^, ^4^, ^5^, ^6^ The risk of esophageal cancer after HNSCC treatment is an 8‐fold to 22‐fold greater than in the general population.^7^, ^8^, ^9^ These SPTs are often diagnosed in advanced stages, which lead to a very low 5‐year survival rate for affected patients.^6^, ^10^, ^11^, ^12^ The prevalence of esophageal‐SPTs in patients with HNSCC is estimated to range from 0% to 22%.^13^

The occurrence of esophageal‐SPTs in patients with HNSCC is often explained by field cancerization of the entire upper aerodigestive tract.^14^, ^15^ The theory of field cancerization states that the mucosal field around the index tumor possesses subtle histologic and genetic changes that increase the risk of synchronous and metachronous malignancies. These subtle tissue changes are thought to be the effect of exposure to accumulating carcinogens (eg, alcohol and tobacco).^10^

Early diagnosis and treatment of an esophageal‐SPT may improve the overall outcome of patients with HNSCC.^5^, ^10^, ^16^ It has even been suggested that its treatment will affect patient survival more than the index HNSCC tumor.^5^ Esophageal carcinomas can remain asymptomatic for a long time during development. A result of this is that many patients’ SPTs only seek medical attention when the tumor is in advanced stages development.^17^ Routine screening of the esophagus in the work‐up and follow‐up of patients with HNSCC could potentially detect more early stage esophageal‐SPTs.^18^, ^19^, ^20^

The diagnosis of esophageal‐SPTs may impact the management of both tumors.^13^ Early stage esophageal‐SPTs may benefit from less invasive endoscopic resection, which can be performed without compromising the treatment of the HNSCC.^21^ However, advanced esophageal‐SPTs are often diagnosed metachronously and will typically be managed by chemoradiotherapy and surgery.^22^ The treatment of the index HNSCC could also hinder that of the esophageal cancer due to treatment sequelae or restrictions to therapeutic options. When possible, personalized treatment should be focused on both tumors.^22^, ^23^

Endoscopic techniques to screen the esophagus have undergone major improvements over the last decades.^10^ White‐light endoscopy is deemed to be insufficient for the detection of superficial cancerous lesions in asymptomatic patients.^9^, ^10^ However, studies with image‐enhanced endoscopy, which includes Lugol's stain, have shown very promising results. Lugol's stain isolates abnormal “mucosal islands” within otherwise normal esophageal tissue, enabling targeted biopsy.^9^ Lugol chromoendoscopy has a high diagnostic accuracy. When combined with narrow band imaging (NBI), it is reported to have a sensitivity of 94.7% and a specificity of 90.4% to detect early stage esophageal lesions.^24^, ^25^

Based on these results, many clinics in Asia implemented esophageal‐SPT screening in patients with HNSCC.^10^ Recently, the French Society of Otorhinolaryngology recommended routine flexible white‐light esophageal endoscopy in the workup of patients with oropharyngeal and hypopharyngeal HNSCC or chronic alcohol use.^13^ The addition of Lugol's stain was recommended. They also suggested to perform routine screening for metachronous esophageal‐SPTs in the follow‐up of patients with HNSCC.^9^

Esophageal Lugol chromoendoscopy is not widely used in the management of patients with HNSCC in the Western world. We performed a systematic review on studies that used Lugol chromoendoscopy to detect esophageal‐SPTs in patients with HNSCC. Our main objective was to investigate the yield of Lugol chromoendoscopy for patients with head and neck cancer in general, but also for specific head and neck sub‐locations. A second aim was to investigate whether current data from non‐Asian patient populations provide enough evidence to justify Lugol chromoendoscopy screening for esophageal‐SPTs in patients with HNSCC in the Western world.

## 2 MATERIALS AND METHODS

### 2.1 Literature search and selection criteria

We searched the Embase, Medline (including PubMed), Web of Science, Cochrane, and Google Scholar databases for relevant studies. The search was performed in April 2017 without a limit on publication date. The following keywords were used for the search: “second/multiple primary tumor,” “esophageal cancer,” and “head and neck cancer.” We limited our search to studies written in English and on humans. Duplicate studies were removed. The remaining citations were reviewed (by OB) bases on title and abstract and in second stage on full text. We included studies that investigated the use of Lugol chromoendoscopy to detect esophageal SPTs in patients with HNSCC. We excluded studies primarily designed as case reports or reviews. The next paragraph presents our full electronic search strategy for the Embase database (see e‐content 1 in the Supplement for full search strategy).

(“second cancer”/exp OR “multiple cancer”/de OR (((Metachronous OR Synchronous OR Second* OR Multiple OR double OR triple OR quadruple OR quintuple OR subsequen* OR Simultan*) NEAR/6 (tumo* OR primary OR malignan* OR carcin* OR neoplas* OR cancer*))):ab,ti) AND (“esophagus tumor”/exp OR “esophagus”/exp OR “esophagus examination”/exp OR (esophag* OR oesophag* OR (upper NEXT/3 (aerodigest* OR digest*))):ab,ti) AND (“head and neck tumor”/exp OR “larynx tumor”/exp OR ((“head”/exp OR neck/exp) AND “primary tumor”/de) OR (((lip OR mouth OR oral OR nose OR nasal OR tongue OR tonsil OR nasopharyn* OR oropharyn* OR hypopharyn* OR pharyn* OR laryn* OR head OR neck) NEAR/10 (tumo* OR primary OR malignan* OR carcin* OR neoplas* OR cancer* OR primar*))):ab,ti) AND [english]/lim NOT ([animals]/lim NOT [humans]/lim).

### 2.2 Assessment of study quality

The methodological quality and risk of bias of the selected Lugol chromoendoscopy screening studies was tested (by OB) with the methodological index for non‐randomized studies (MINORS).^26^ Its relevance to the current topic was determined using a 3‐criterion checklist, including (1) impact factor of publishing journal and thus an indication of quality of peer review, (2) data on the prevalence of esophageal‐SPT per head and neck sub‐location, and (3) clarity of the text (Table 1). The total score of both the MINORS scale and relevance criteria was used as a quality score. Based on this score, the quality was classified as low (total score ≤ 10 points), medium (total score 11‐14 points), or high (total score ≥ 15 points). Studies of medium and high quality were included for further analysis and low‐quality studies were excluded.

**Table 1:** Relevance criteria

	Score		
Criteria	0	1	2
Impact factor	<2	2‐3.9	≥4
Sub‐location	No	‐	Yes
Text clarity	Low	Medium	High

**Table 2:** Characteristics and MINORS + relevance scores of all 23 Lugol chromoendoscopy screening studies

					Score			
Authors	Country	Year	Design	*N*	MINORS	Relevance	Total	Quality
**Included studies**								
Gong et al^29^	Korea	2016	Pro	458	10	5	15	High
Wang CH et al^25^	Taiwan	2014	Pro	294	11	3	14	Medium
Wang, Wang, et al^22^	Taiwan	2013	Pro	180	9	5	14	Medium
Ikawa et al^30^	Japan	2012	Pro	171	8	4	12	Medium
Wang, Lee, et al^31^	Taiwan	2011	Pro	315	11	5	16	High
Morimoto et al^21^	Japan	2010	Pro	64	7	4	11	Medium
Fukuhara et al^32^	Japan	2010	Pro	157	8	4	12	Medium
Lee et al^33^	Taiwan	2009	Pro	44	11	4	15	High
Boller et al^34^	Switzerland	2009	Pro	40	11	3	14	Medium
Dubuc et al^35^	France	2006	Pro	393	10	3	13	Medium
Hashimoto et al^36^	Brazil	2005	Pro	326	10	4	14	Medium
Muto, Nakane, et al^37^	Japan	2002	Pro	78	9	4	13	Medium
Tanabe et al^38^	Japan	2001	Retro	134	8	3	11	Medium
Fukuzawa et al^39^	Japan	1999	Pro	56	7	4	11	Medium
Horiuchi et al^40^	Japan	1998	Retro	676	7	4	11	Medium
**Excluded studies**								
Laohawiriyakamol et al^41^	Thailand	2014	Pro	89	10	0	10	Low
Komínek et al^42^	Czech R.	2013	Pro	132	9	0	9	Low
Chow et al^43^	China	2009	Retro	118	7	2	9	Low
Muto, Hironaka, et al^44^	Japan	2002	Retro	389	6	1	7	Low
Tincani et al^45^	Brazil	2000	Pro	60	7	0	7	Low
Ina et al^46^	Japan	1994	Pro	127	7	2	9	Low
Chisholm et al^47^	China	1992	Pro	37	7	0	7	Low
Shiozaki et al^48^	Japan	1990	Pro	178	7	2	9	Low

Abbreviations: MINORS, methodological index for non‐randomized studies; *N*, number of patients with head and neck cancer included; Pro, prospective; Retro, retrospective; Year, year of publication.

### 2.3 Data extraction

Data from all included studies were extracted onto record forms (by OB) and results were summarized as a Preferred Reporting Items for Systematic Reviews and Meta‐Analyses check list and flowchart.^27^ The total prevalence of diagnosed esophageal‐SPTs was recorded as primary outcome. An esophageal‐SPT was defined as an esophageal lesion classified as category 4 and 5: high‐grade dysplasia (HGD) or carcinoma. When possible 3 secondary outcomes were recorded: (1) the SPT prevalence per sub‐location of the index head and neck tumor (oral cavity, oropharynx, hypopharynx, larynx, nasopharynx, and other) and per tumor stage (0‐4) of the index tumor; (2) whether the SPTs were diagnosed synchronously (≤6 months after diagnosis of index tumor, in some cases simultaneously) or metachronously (>6 months after diagnosis of index tumor); and (3) in which stage of development the SPTs was according to the Vienna classification of gastrointestinal epithelial neoplasia.^28^ Finally, first author, country of study population, year of publication, study design, and population size were also recorded.

### 2.4 Statistical analysis

Data were reported as counts and percentages. The SPT prevalence was calculated for each study as the total number of detected SPTs divided by the total population that was screening in the particular study. In studies where the SE was not reported, we calculated it from the prevalence using the following formula: SE = √*p*(1 − *p*)/*n*; in which *p* is the prevalence and *n* is the total number of patients with ESCC that were screened for head and neck SPTs. Review Manager software (version 5.3) was used for meta‐analysis. Random effects model was used to calculate the pooled prevalence. *I* ^2^ was used to evaluate the level of heterogeneity between studies. Subgroup analyses were performed for specific head and neck cancer sub‐locations.

## 3 RESULTS

### 3.1 Study selection, quality assessment, and characteristics

**Figure 1:**
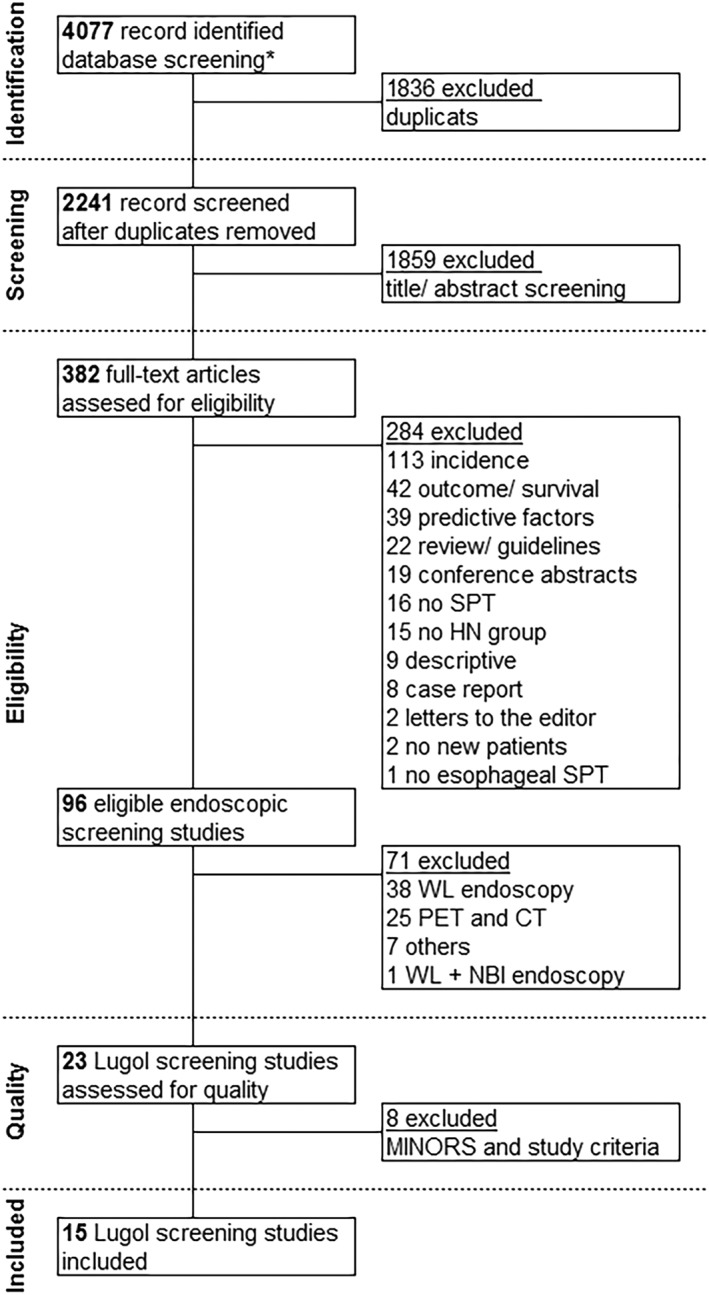
Study selection process. *, Embase, Medline, Web of Science, Cochrane, and Google Scholar; HN, head and neck; MINORS, methodological index for non‐randomized studies; NBI, narrow band imaging; SPT, multiple primary tumor; WL, white light

Results of our search query for eligible qualitative Lugol chromoendoscopy screening studies are presented in Figure 1. The search identified 4077 citations. After removing duplicates 2241 citations were reviewed. Based on review of title and abstract, 1859 citations were excluded. The remaining 382 studies were reviewed for their eligibility by reviewing the full text. This revealed 96 studies that screened a population of patients with HNSCC for esophageal SPTs. Reasons for exclusion of other studies are mentioned in Figure 1. Review of the 96 screening studies resulted in the selection of 23 Lugol chromoendoscopy screening studies (Table 2).^21^, ^22^, ^25^, ^29^, ^30^, ^31^, ^32^, ^33^, ^34^, ^35^, ^36^, ^37^, ^38^, ^39^, ^40^, ^41^, ^42^, ^43^, ^44^, ^45^, ^46^, ^47^, ^48^ Most other screening studies were performed with only white‐light endoscopy (eg, triple‐endoscopy) or with the use of PET/CT.

The combined quality score of the MINORS and relevance criteria qualified 15 studies as medium or high quality and these were included in the present review.^21^, ^22^, ^25^, ^29^, ^30^, ^31^, ^32^, ^33^, ^34^, ^35^, ^36^, ^37^, ^38^, ^39^, ^40^ The remaining studies of low quality were excluded. The methodological quality assessment using MINORS resulted in scores ranging from 6 to 11 points (median 8 and maximal possible score 16). The relevance criteria score ranged from 0 to 5 points (median 3 and maximal possible score 6).

Twelve of the studies included (80%) were performed in Asia (Korea, Japan, and Taiwan) and the remaining 3 in Switzerland, France, and Brazil. Nine studies were performed within the last decade and all studies within the last 2 decades. Most studies collected data prospectively (13, 87%). The study populations ranged from 40 to 676 patients (median 171) and the total number of patients was 3386. All studies used similar methods by applying 10‐40 mL of 0.8%‐3.0% Lugol's solution on the esophageal mucosa.

### 3.2 Prevalence

**Figure 2:**
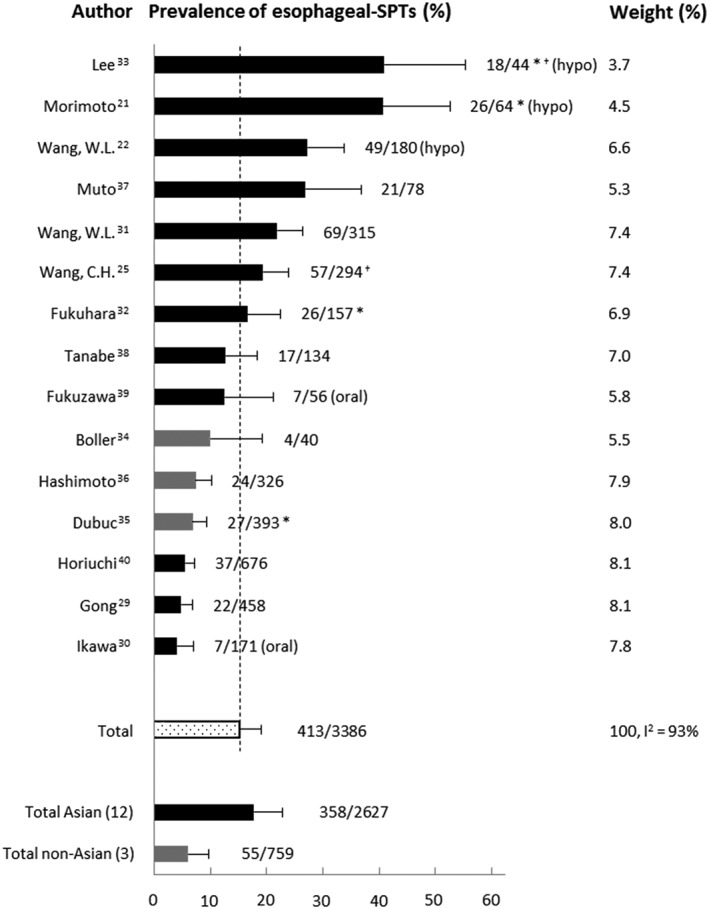
Overview of prevalence of esophageal‐SPTs of 15 Lugol chromoendoscopy screening studies. Error bars represent 95% confidence intervals. *, both synchronous and metachronous screening; ^†^, transnasal Lugol chromoendoscopy; hypo, study included only patients with hypopharyngeal tumors; oral, study included only patients with oral cavity tumors; SPT, multiple primary tumor

The average prevalence of esophageal‐SPTs in patients with HNSCC of the 15 included studies was 15.2% (413 of 3386, 95% CI: 11.4‐19.0) (Figure 2). The 3 studies with the highest prevalence included only or mostly patients with a hypopharyngeal index tumor.^21^, ^22^, ^33^ Two Japanese studies only included patients with oral cavity tumors.^30^, ^39^ The average esophageal‐SPT prevalence of the 12 Asian studies was 17.7% (358 of 2627, 95% CI: 12.7‐22.7). This was higher than the average of the three non‐Asian studies: 6.0% (55 of 759, 95% CI: 2.3‐9.7).

### 3.3 Prevalence per sub‐location

**Figure 3:**
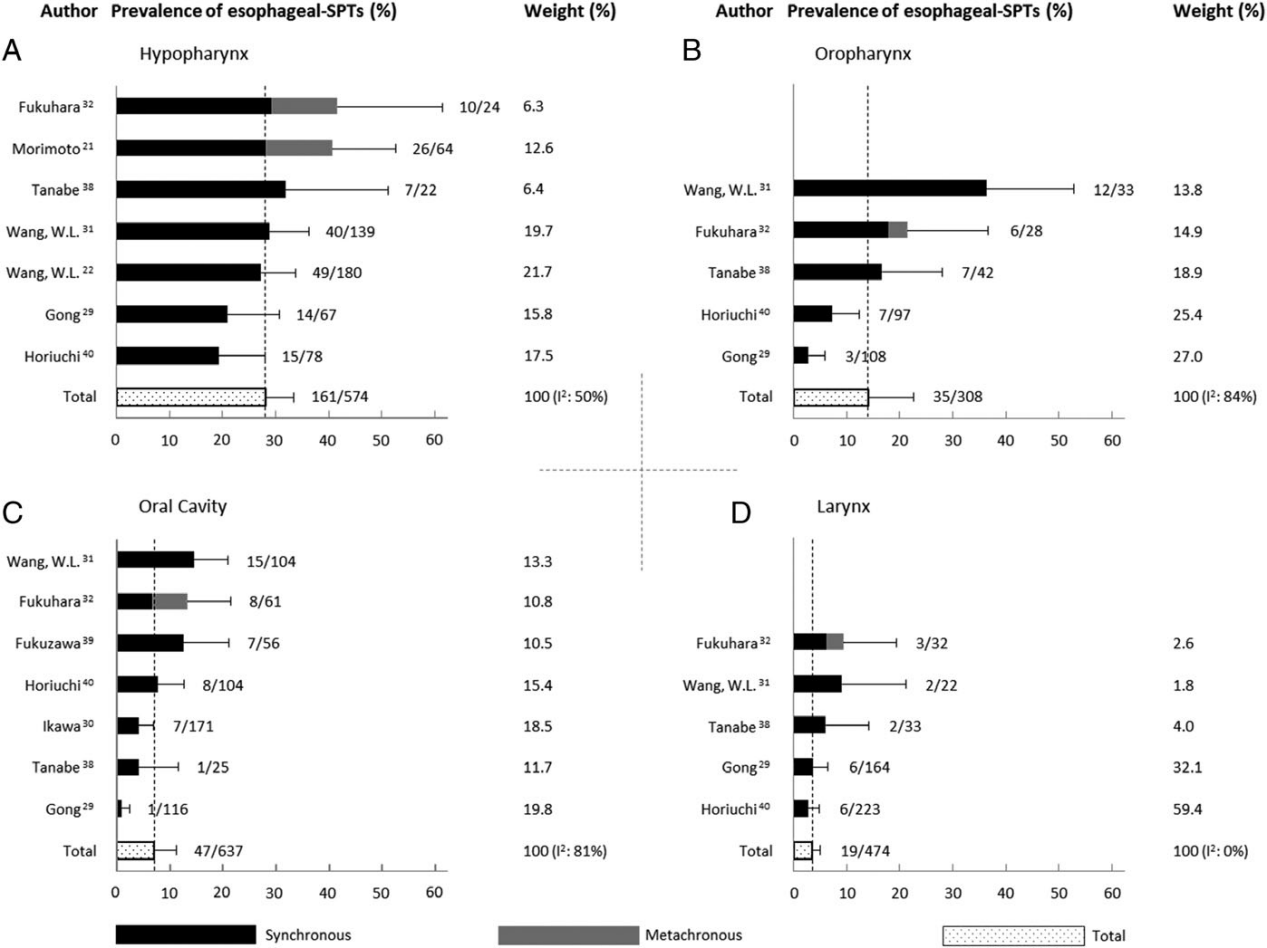
Overview of prevalence of esophageal‐second primary tumors (SPTs) per sub‐location of index head and neck squamous cell carcinoma: A, hypopharynx; B, oropharynx; C, oral cavity; and D, larynx. Nine Asian studies with sub‐location specific data. Fukuhara et al and Morimoto et al screened both synchronously and metachronously. Error bars represent 95% confidence intervals

Nine Asian studies reported data of esophageal‐SPTs per sub‐location of the index HNSCC (Figure 3).^21^, ^22^, ^29^, ^30^, ^31^, ^32^, ^38^, ^39^, ^40^ The prevalence of esophageal‐SPTs was the highest in patients with hypopharyngeal index tumors, followed by patients with oropharyngeal, oral cavity, and laryngeal and nasopharyngeal tumors. The average prevalence of esophageal lesions in patients with hypopharyngeal tumors of seven studies was 28.0% (161 of 574, 95% CI: 22.5‐33.5). Five studies reported an average of 14.0% (35 of 308, 95% CI: 5.4‐22.5) esophageal‐SPTs in patients with oropharyngeal tumors. The diagnostic yield of Lugol chromoendoscopy in patients with oral cavity tumors was 7.2% (47 of 637, 95% CI: 3.2‐11.2). For patients with laryngeal index tumors the rate of esophageal‐SPTs was 3.4% (19 of 474, 95% CI: 1.8‐5.4). Four studies reported only 2 esophageal‐SPTs in 109 patients with nasopharyngeal tumors and none were found in patients with other index tumors (eg, glandular tumors).

### 3.4 Time to diagnosis

Most studies only performed endoscopic screening of the esophagus in the work‐up of the index HNSCC tumor and thus only diagnosed synchronous, or even simultaneous esophageal‐SPTs. Four studies performed both synchronous and metachronous esophageal endoscopies.^21^, ^32^, ^33^, ^35^ Morimoto et al performed at diagnosis of the HNSCC and annually during follow‐up.^21^ Eighteen (69.2%) of all SPTs were diagnosed synchronously and 8 (30.8%) metachronously. Fukuhara et al found a similar distribution between synchronously diagnosed SPTs (*n* = 17 [60.7%]) and those that were diagnosed metachronously (*n* = 9 [32.1%]).^32^ The two other studies also metachronous endoscopies, but did not separately mention the synchronous or metachronous diagnostic yield of Lugol chromoendoscopy.^33^, ^35^

### 3.5 Vienna classification

Eight studies differentiated between esophageal‐SPTs classified as category 4 (HGD) or 5 (carcinoma).^21^, ^25^, ^29^, ^31^, ^33^, ^34^, ^35^, ^36^ The remaining studies either did not mention the category or only mentioned category 5. Almost half of all esophageal‐SPTs found in these 8 studies (48.6%, range 22.2‐100.0) were category 4 lesions, HGD. That was approximately the same for Asian (43.3%, *n* = 5) and non‐Asian (57.4%, *n* = 3) studies. Three of these 7 studies also differentiated the esophageal carcinoma's in low‐stage (stages I and II) and high‐stage (stages III and IV) esophageal tumors.^21^, ^29^, ^31^ Their combined data show that 53.9% (41/76) of all esophageal carcinoma's were classified as low stage and 46.1% (35/76) as high stage.

### 3.6 Prevalence per index tumor stage

Three Asian studies also reported the prevalence of esophageal‐SPTs in patients with HNSCC per tumor stage of the index tumor.^21^, ^29^, ^39^ There were a total of 5 (3.1%, 95% CI: 0.3‐5.8) esophageal‐SPTs in 150 patients with stage I HNSCC, 28.8% (95% CI: −5.7 to 63.3) esophageal‐SPTs in patients with stage II HNSCC, 5.34% (95% CI: 1.1‐9.6) esophageal‐SPTs in stage III HNSCC, and 22.5% (95% CI: −2.3 to 47.3) in patents with a stage IV index HNSCC.

## 4 DISCUSSION

To the best of our knowledge, this is the first systematic review on the diagnostic yield of Lugol chromoendoscopy for esophageal SPTs in patients with HNSCC. Our main findings show that on average, 15% of the patients with primary HNSCC that underwent Lugol chromoendoscopy were diagnosed with an esophageal‐SPT. We found that the prevalence was the highest for patients with hypopharyngeal index tumors.

There is a large discrepancy between the prevalence of esophageal‐SPTs in patients with HNSCC found with Lugol chromoendoscopy screening (15%, 95% CI: 11‐19) and the prevalence of retrospective non‐screening studies (1%‐6%).^6^, ^7^, ^49^, ^50^, ^51^, ^52^, ^53^ This was also noted by Wang, Lee, et al.^31^ This discrepancy could indicate that without an active screening program, esophageal‐SPTs are underdiagnosed in patients with HNSCC.^7^ Multiple studies state that the occurrence of esophageal‐SPTs negatively influences patient survival, especially in patients with advanced esophageal‐SPTs.^23^, ^54^, ^55^, ^56^ Some researchers even claim that SPTs are the leading cause of treatment failure and death in patients with HNSCC.^31^

The hypopharynx, and in particular involvement of the piriform sinus, is a well‐known risk factor for the development of esophageal‐SPTs.^57^, ^58^, ^59^, ^60^ The results from the present review also SPTs underlined this. Wang WL et al compared two hypopharyngeal HNSCC cohorts: before and after implementing pretreatment Lugol chromoendoscopy esophagus screening.^22^ Active esophageal screening tripled the amount of diagnosed esophageal‐SPTs (5.3% vs 15.3%). The present study also found esophageal‐SPTs in 11% of patients with oropharyngeal cancer, which is also a known sub‐location to be at risk factor for the development of an esophageal‐SPT.^60^, ^61^ However, the 2 largest studies in this review with specific oropharynx data by Horiuchi et al and Gong et al found relatively low prevalence of esophageal‐SPTs (7.2% and 2.8%) in this subgroup of patients.

The finding that up to a third of all esophageal‐SPTs found in the studies by Morimoto et al and Fukuhara et al were diagnosed metachronously during follow‐up could indicate that the results of the other synchronous studies underestimate the true prevalence of esophageal‐SPTs.^21^, ^32^ It is also an indication that esophageal screening of patients with HNSCC should also be performed in the follow‐up of the index tumor. However, the optimal esophageal screening schedule has yet to be defined.

Approximately 50% of the esophageal lesions found in this review were classified as HGD. Of the remaining lesions classified as invasive carcinoma, about half were early stage. This is similar to the findings from other researchers.^22^, ^29^, ^38^ Wang, Wang, et al showed that an active screening protocol diagnosed more HGD lesions and early stage carcinomas, which significantly reduced the mortality rate of affected patients.^22^ This is possibly the result of adjustments of the treatment strategy aimed at treating 2 instead of 1 tumor and less invasive endoscopic treatment of the esophageal lesions. Multiple studies claim that treatment of the esophageal‐SPT increases the survival, especially in patients with early stage tumors.^23^, ^54^, ^55^, ^56^

Five of the included studies in this review used NBI in addition to Lugol chromoendoscopy.^22^, ^25^, ^29^, ^31^, ^33^ Wang CH et al concluded that this combination of both techniques has the highest diagnostic accuracy to detect esophageal lesions: a sensitivity of 95%, a specificity of 90%, and an accuracy of 91% (95% CI: 88‐94).^25^ Several other researchers have investigated the use of full‐body ^18^F‐FDG‐PET/CT. They reported a considerably lower diagnostic esophageal‐SPT yield that ranged from 0.43% to 4.85%.^62^, ^63^, ^64^, ^65^, ^66^ As Kondo et al also mentioned that PET/CT seems to be an inferior technique for detection of esophageal‐SPTs because it is not sensitive for early tumors.^62^

Two of the studies included in this review performed transnasal Lugol chromoendoscopy.^25^, ^33^ Tumor‐related airway obstruction or postradiation trismus sometimes make the oropharyngeal passage difficult to reach with conventional endoscopes. The transnasal route bypasses this problem. Transnasal Lugol chromoendoscopy has the additional advantage that it can be performed in unsedated patients and that it even has a higher completion rate than conventional endoscopy.^67^

The prevalence of SPTs after HNSCC in the existing literature varies greatly geographically. In Asia, second primary gastrointestinal tract malignancies are more common after index HNSCC than in the Western world.^2^ It is thought that Asians have a higher exposure to risk factors such as smoking and alcohol use. Other risk factors such as hot beverage drinking and betel quid chewing and genetic susceptibility have also been suggested to play a part.^68^ As a result, the literature on this topic, including the studies of this review, is mostly from Asian countries. In the present review, only 3 studies were non‐Asian. This prohibits us to draw bold conclusions and extrapolate results on the usefulness of Lugol chromoendoscopy in a non‐Asian patient population, as also stated by Morimoto et al.^21^

Another limitation is the quality of the included studies. As the quality of a review greatly relies on the quality of the included data, we excluded studies of low quality. Although the remaining 15 studies were all similar in methodology and research question, there was some heterogeneity among the studies in the subsites of the index HNSCC tumors that were included. This might have had an influence on the average prevalence of all studies. However, the 4 largest studies (*n* = 326‐676) with the highest weight on the average fortunately included all sub‐locations. A final potential limitation is that the study selection and quality assessment was performed by 1 reviewer. The overall study quality could have benefited from an assessment by 2 independent reviewers.

In conclusion, this review has shown that the prevalence of esophageal SPTs in patients with head and neck cancer is high, especially for patients with a hypopharyngeal and oropharyngeal index tumor. A large percentage of esophageal lesions were found in early stage of development. Literature shows that this group of patients could significantly benefit from dual tumor treatment, resulting in an increased 5‐year survival rate. Based on our results, there appears to be strong evidence to perform Lugol chromoendoscopy screening in an Asian patient population. More screening studies are needed to confirm the same for the Western world, and Lugol chromoendoscopy holds the potential to increase the overall survival rate of patients with head and neck cancer, due a lowered SPT‐specific mortality.

## CONFLICT OF INTEREST

The authors declare that they have no conflicts of interest with the content of this article.

## Supporting information

Supporting Information.
