# Supplementary material for: Early detection of esophageal second primary tumors using Lugol chromoendoscopy in patients with head and neck cancer: A systematic review and meta‐analysis
Source: Head Neck. 2018 Dec 28;41(4):1122–30. doi: 10.1002/hed.25548 (PMC6590301; doi:10.1002/hed.25548)
Supplement: Supplementary file 1 — Supporting Information. [file HED-41-1122-s001.docx]

**Literature search ‘Early detection of esophageal second primary tumors using Lugol chromoendoscopy in patients with head and neck cancer: a systematic review’**

26 April 2017

| Embase.com | 1688 | 1659 |
| --- | --- | --- |
| Medline Ovid | 1122 | 182 |
| Web of science | 1028 | 277 |
| Cochrane CENTRAL | 39 | 3 |
| Google scholar | 200 | 120 |
| **Total** | **4077** | **2241** |

**Embase.com 1688**

('second cancer'/exp OR 'multiple cancer'/de OR (((Metachronous OR Synchronous OR Second* OR Multiple OR double OR triple OR quadruple OR quintuple OR subsequen* OR Simultan*) NEAR/6 ( tumo* OR primary OR malignan* OR carcin* OR neoplas* OR cancer*))):ab,ti) AND ('esophagus tumor'/exp OR 'esophagus'/exp OR 'esophagus examination'/exp OR (esophag* OR oesophag* OR (upper NEXT/3 (aerodigest* OR digest*))):ab,ti) AND ('head and neck tumor'/exp OR 'larynx tumor'/exp OR (('head'/exp OR neck/exp) AND 'primary tumor'/de) OR (((lip OR mouth OR oral OR nose OR nasal OR tongue OR tonsil OR nasopharyn* OR oropharyn* OR hypopharyn* OR pharyn* OR laryn* OR head OR neck ) NEAR/10 (tumo* OR primary OR malignan* OR carcin* OR neoplas* OR cancer* OR primar*))):ab,ti) AND [english]/lim NOT ([animals]/lim NOT [humans]/lim)

**Medline Ovid 1122**

("Neoplasms, Second Primary"/ OR (((Metachronous OR Synchronous OR Second* OR Multiple OR double OR triple OR quadruple OR quintuple OR subsequen* OR Simultan*) ADJ6 ( tumo* OR primary OR malignan* OR carcin* OR neoplas* OR cancer*))).ab,ti,kf.) AND ("Esophageal Neoplasms"/ OR "esophagus"/ OR (esophag* OR oesophag* OR (upper ADJ3 (aerodigest* OR digest*))).ab,ti,kf.) AND ("Head and Neck Neoplasms"/ OR exp "Mouth Neoplasms"/ OR exp "Otorhinolaryngologic Neoplasms"/ OR (((lip OR mouth OR oral OR nose OR nasal OR tongue OR tonsil OR nasopharyn* OR oropharyn* OR hypopharyn* OR pharyn* OR laryn* OR head OR neck ) ADJ10 (tumo* OR primary OR malignan* OR carcin* OR neoplas* OR cancer* OR primar*))).ab,ti,kf.) AND english.la. NOT (exp animals/ NOT humans/)

**Cochrane CENTRAL 39**

((((Metachronous OR Synchronous OR Second* OR Multiple OR double OR triple OR quadruple OR quintuple OR subsequen* OR Simultan*) NEAR/6 ( tumo* OR primary OR malignan* OR carcin* OR neoplas* OR cancer*))):ab,ti) AND ((esophag* OR oesophag* OR (upper NEXT/3 (aerodigest* OR digest*))):ab,ti) AND ((((lip OR mouth OR oral OR nose OR nasal OR tongue OR tonsil OR nasopharyn* OR oropharyn* OR hypopharyn* OR pharyn* OR laryn* OR head OR neck ) NEAR/10 (tumo* OR primary OR malignan* OR carcin* OR neoplas* OR cancer* OR primar*))):ab,ti)

**Web of science 1028**

TS=(((((Metachronous OR Synchronous OR Second* OR Multiple OR double OR triple OR quadruple OR quintuple OR subsequen* OR Simultan*) NEAR/5 ( tumo* OR primary OR malignan* OR carcin* OR neoplas* OR cancer*)))) AND ((esophag* OR oesophag* OR (upper NEAR/2 (aerodigest* OR digest*)))) AND ((((lip OR mouth OR oral OR nose OR nasal OR tongue OR tonsil OR nasopharyn* OR oropharyn* OR hypopharyn* OR pharyn* OR laryn* OR head OR neck ) NEAR/9 (tumo* OR primary OR malignan* OR carcin* OR neoplas* OR cancer* OR primar*)))) ) AND LA=(english)

**Google scholar**

"Metachronous|Synchronous|Second|Multiple|Simultaneous tumor|cancer|primary" esophagus|esophageal|oesophagus|oesophageal "lip |mouth|oral|pharyngeal|larygeal|head|neck tumor|malignancy|carcinma|neoplasms|cancer"
